# Toward Responsible Artificial Intelligence in Long-Term Care: A Scoping Review on Practical Approaches

**DOI:** 10.1093/geront/gnab180

**Published:** 2021-12-06

**Authors:** Dirk R M Lukkien, Henk Herman Nap, Hendrik P Buimer, Alexander Peine, Wouter P C Boon, Johannes C F Ket, Mirella M N Minkman, Ellen H M Moors

**Affiliations:** Vilans Centre of Expertise for Long-Term Care, Utrecht, The Netherlands; Copernicus Institute of Sustainable Development, Utrecht University, Utrecht, The Netherlands; Vilans Centre of Expertise for Long-Term Care, Utrecht, The Netherlands; Human Technology Interaction, Eindhoven University of Technology, Eindhoven, The Netherlands; Vilans Centre of Expertise for Long-Term Care, Utrecht, The Netherlands; Copernicus Institute of Sustainable Development, Utrecht University, Utrecht, The Netherlands; Copernicus Institute of Sustainable Development, Utrecht University, Utrecht, The Netherlands; Medical Library, Vrije Universiteit, Amsterdam, The Netherlands; Vilans Centre of Expertise for Long-Term Care, Utrecht, The Netherlands; TIAS School for Business and Society, Tilburg University, Tilburg, The Netherlands; Copernicus Institute of Sustainable Development, Utrecht University, Utrecht, The Netherlands

**Keywords:** Ethics, Intelligent technology, Responsible innovation

## Abstract

**Background and Objectives:**

Artificial intelligence (AI) is widely positioned to become a key element of intelligent technologies used in the long-term care (LTC) for older adults. The increasing relevance and adoption of AI has encouraged debate over the societal and ethical implications of introducing and scaling AI. This scoping review investigates how the design and implementation of AI technologies in LTC is addressed responsibly: so-called responsible innovation (RI).

**Research Design and Methods:**

We conducted a systematic literature search in 5 electronic databases using concepts related to LTC, AI, and RI. We then performed a descriptive and thematic analysis to map the key concepts, types of evidence, and gaps in the literature.

**Results:**

After reviewing 3,339 papers, 25 papers were identified that met our inclusion criteria. From this literature, we extracted 3 overarching themes: user-oriented AI innovation; framing AI as a solution to RI issues; and context-sensitivity. Our results provide an overview of measures taken and recommendations provided to address responsible AI innovation in LTC.

**Discussion and Implications:**

The review underlines the importance of the context of use when addressing responsible AI innovation in LTC. However, limited empirical evidence actually details how responsible AI innovation is addressed in context. Therefore, we recommend expanding empirical studies on RI at the level of specific AI technologies and their local contexts of use. Also, we call for more specific frameworks for responsible AI innovation in LTC to flexibly guide researchers and innovators. Future frameworks should clearly distinguish between RI processes and outcomes.

Artificial intelligence (AI) is widely positioned and predicted to become a key element of intelligent technologies that are used in the long-term care (LTC) for older adults (Ho, 2020; Rubeis, 2020). AI technologies are machine-based systems that can, for a given set of human-defined objectives, make predictions, recommendations, or decisions influencing real or virtual environments (Yeung, 2020). They perform functions such as image, speech and pattern recognition, and natural language processing which are normally associated with the human brain (McCarthy et al., 2006). AI technologies can be driven by both *pre-programmed, rule-based* algorithms that capture and embody the knowledge of human experts in a specialized domain (Lucas & Van Der Gaag, 1991; Miller et al., 1982) and *self-learning, case-based* algorithms that independently learn to execute tasks and improve on the basis of machine learning on historical, exemplary data (Aamodt & Plaza, 1994; Jordan & Mitchell, 2015; LeCun et al., 2015; Samuel, 1959). Accordingly, AI technologies are designed to operate with varying levels of autonomy (Yeung, 2020).

In LTC, AI is said to enable and improve an increasing variety of intelligent technologies such as remote monitoring systems, recommendation and decision support software, social robots, and virtual assistants that interact with older adults and their caregivers on a daily basis. One widespread expectation of AI is that it allows such technologies to learn about their environment and adapt to changing contexts of action (Dermody & Fritz, 2019; Ho, 2020; Mukaetova-Ladinska et al., 2020). For example, through AI, camera-based monitoring systems can learn to classify activities such as lying, sitting, standing, and walking. They can also predict the ease and the amount of time a person spends getting out of bed or the risk of events such as a fall (Cardinaux et al., 2011; Luo et al., 2018). Besides, sensor-based monitoring systems can track older adults’ walking speed and daily presence in different rooms. AI can enable such technologies to identify unusual variations in movements and activities that may indicate cognitive and functional decline. By sending automated alerts or behavioral suggestions to the older person and/or their (in)formal caregivers, AI-based monitoring technologies can facilitate timely care and potentially preventing further deteriorations (J. A. Kaye et al., 2011; Zwierenberg et al., 2018). This can help to delay or avoid nursing home admission. Further, research indicates that older adults and their informal caregivers experience a greater sense of safety and reduced subjective stress when using automated monitoring systems at home (Ho, 2020; Pol et al., 2016; Zwierenberg et al., 2018). Nonetheless, it is also widely recognized that the use of remote monitoring technologies in home-based and institutional settings poses risks related to things such as privacy, dignity, autonomy, trust, equal access, and the disruption of care (Berridge et al., 2019; Chung et al., 2016; Grigorovich & Kontos, 2020; Zwijsen et al., 2011).

Despite its promises and benefits, the increasing relevance and adoption of AI in LTC and other domains of society has encouraged debate over the societal and ethical implications of introducing and scaling AI (Good, 1966; Morley et al., 2019; Rubeis, 2020; Russell et al., 2015; Tsamados et al., 2021; Zuboff, 2015). It is recognized that the use of AI can lead to more effective, efficient, and sometimes more transparent decisions than those made by human beings. However, it can also lead to harmful consequences such as undermining of people’s privacy, autonomy, and self-determination, while exacerbating bias, opacity, and mass unemployment (Burrell, 2016; Crawford & Calo, 2016; Frey & Osborne, 2017; Helbing et al., 2018; O’Neil, 2016; Obermeyer et al., 2019; Zou & Schiebinger, 2018). The use of AI in technologies in LTC may exacerbate negative effects of technologies such as the problematization, medicalization, and stigmatization of old age beside the depersonalization and dehumanization of care (Rubeis, 2020). Carefully balancing the promises and benefits of AI with its risks and downsides calls for *responsible innovation* (RI), which requires innovators, users, and other stakeholders to have a critical look at the social and ethical consequences of AI technologies for older people, their environment, and society as a whole.

Recent years have seen a growing prevalence of frameworks, principles, and guidelines to inform responsible AI innovation. Here, we have opted for the term “responsible” AI, but this topic can also be phrased as “ethical,” “trustworthy,” or “sustainable” AI. Studies that have dealt with responsible AI frameworks emphasize the importance of high-level principles such as transparency, justice, fairness, and nonmaleficence (Fjeld et al., 2020; Hagendorff, 2020; Jobin et al., 2019). Far less attention has been paid to the *implementation and impact of such principles in the actual design and implementation of AI in practice.* This could be problematic because high-level principles leave much room for interpretation as to how they can be practically applied in specific contexts of use such as LTC (Floridi, 2019; Hagendorff, 2020; Jobin et al., 2019). It has thus remained unclear how responsible AI principles unfold their expected relevance in actual practices of AI design and implementation in LTC. In this paper, we present the results of a scoping literature review to better understand the current state of knowledge on how RI is addressed in the design and implementation of AI technologies in LTC that are used by older adults and/or their formal and informal caregivers.

## Research Design and Methods

Scoping reviews are a specific type of literature review aimed at mapping the existing literature in a broad field of interest. These are suitable to describe the current state-of-science in a given research area and identify key lessons and knowledge gaps that could be studied further (Arksey & O’Malley, 2005; Rumrill et al., 2010). Our approach is based on the reporting guidelines established by Tricco et al. (2018; see Supplementary Section A). In the following, we describe the search strategy, the process of selecting papers that were included in this scoping review, and the protocols that were followed to synthesize results.

### Search Strategy

In multiple iterations, we developed a search query that covers the set of terms related to three core search concepts defined by our research aim: (a) LTC, (b) AI and technologies in LTC that are potentially driven by AI, and (c) RI (see Supplementary Section B). Five databases were searched from inception (by D. R. M. Lukkien and J. C. F. Ket): PubMed (up to 17 June 2020), Clarivate Analytics/Web of Science Core Collection and Elsevier/Scopus (up to 14 July 2020), Ebsco/APA PsycINFO (up to 21 August 2020), and Ebsco/CINAHL (up to 8 September 2020). The search was limited to English language papers and no time frame restrictions were made. The systematic search identified 4,791 records. In addition, 16 records were identified through citation chaining and associative searches with limited search terms in the electronic databases ACM Digital Library and IEEE Xplore. After removing duplicates, 3,339 papers entered the screening phase.

### Selection of Papers

All authors were involved at the beginning, middle, and end of the screening process to ensure consistency and investigator triangulation. We defined and refined inclusion criteria for each of the core concepts in our search before and throughout the iterative screening process:

(1) LTC: eligible papers address technological systems or services that are (to be) used by older adults who receive LTC, and/or used by their formal and informal caregivers. By LTC, we mean the assistance given over an extended period of time to people who, as a result of aging and related conditions such as dementia, experience inabilities to perform tasks associated with everyday living (Kane et al., 1998; H. S. Kaye et al., 2017). This can be both formal and informal care in institutionalized, community- or home-based care settings.
(2) AI: eligible papers provide information about the (semi)autonomous decision-making capabilities of the addressed technologies, that is, about the data-processing mechanisms that enable them to carry out certain tasks independently. Responsible AI innovation can only be properly assessed if clear explanations are provided about the role of AI in the article (Hagendorff, 2020).
(3) RI: eligible papers report on recommendations for decisions in practice to foster the responsible design and/or implementation of AI technologies in LTC. For instance, eligible papers describe how certain measures relating to design or implementation of AI technologies contribute to the ethical acceptability, sustainability, and/or social desirability of these technologies (Von Schomberg, 2013) or to their compliance with responsible AI principles like transparency, justice, and fairness (Jobin et al., 2019). Papers are excluded when they question *if* AI technologies can be responsibly used in LTC without discussing *how* they can be responsibly designed or implemented. Papers are also excluded when they discuss which RI issues should be addressed in context of a particular AI technology, without providing clues on how to address these issues at the level of the technology’s design or implementation. Further, papers are excluded if they solely assess the accuracy, usability, or acceptability of technologies.

**Figure 1.**
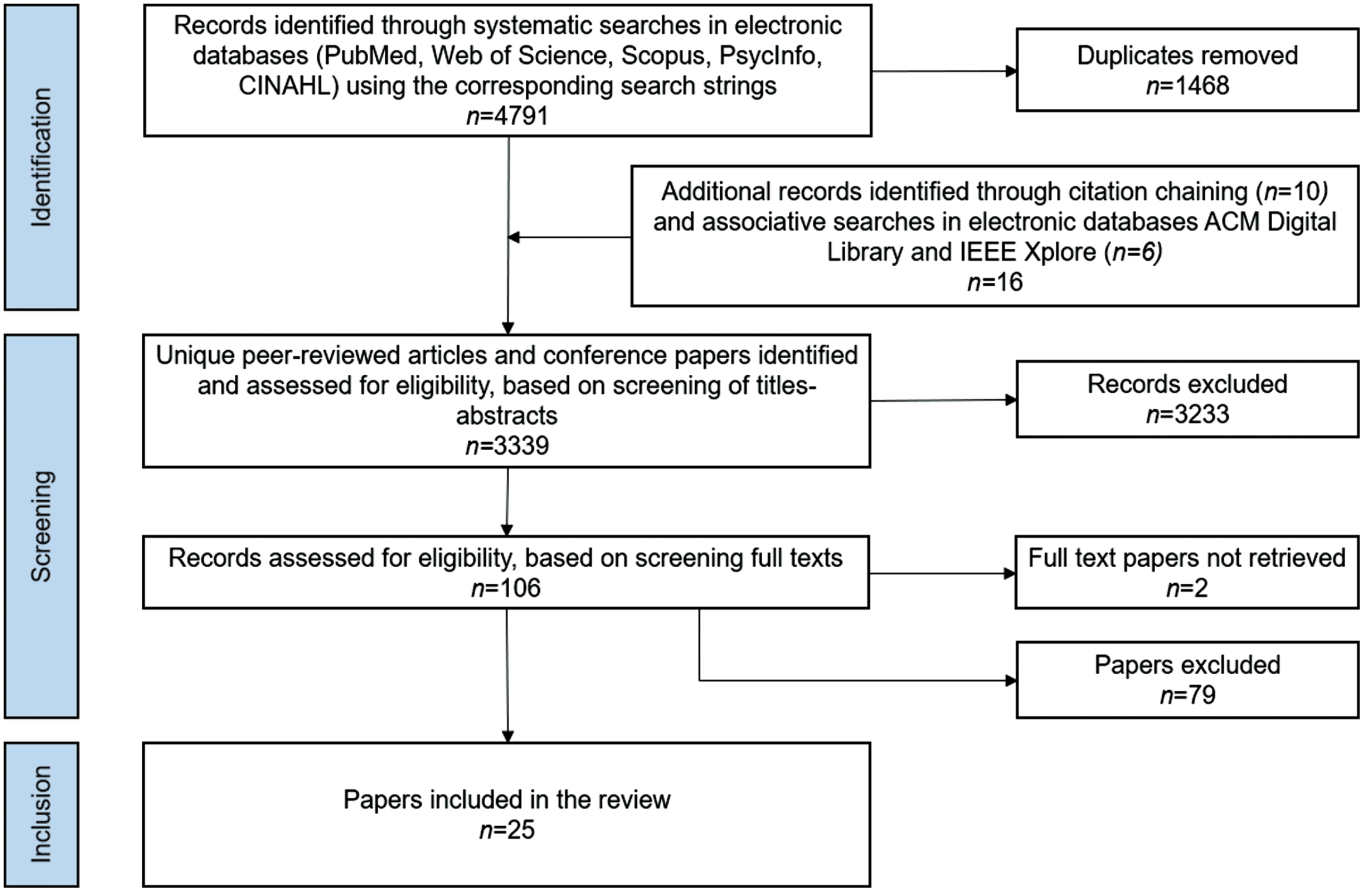
Flowchart of our retrieval process.

The review comprised two stages. To minimize subjective biases, the authors acting as literature reviewers performed each stage independently from each other. First, a title and abstract screening was performed (by D. R. M. Lukkien and H. P. Buimer) to select papers that met all three main inclusion criteria. When one reviewer had doubt on compliance with one or more criteria, or if there was any disagreement between the reviewers, they discussed the article orally, or if necessary, together with a third reviewer (H. H. Nap), to reach consensus. After exclusion of duplicates following the preliminary screening, 106 papers were subject to full-text reading. In a second round of full-text screening (by D. R. M. Lukkien and H. H. Nap), records that discussed any of the three core search concepts only marginally and that made an insufficient link *between* the three core search concepts were excluded. For papers by the same authors and with similar content, only the most recent peer-reviewed article was included. Finally, 25 papers were selected for the review. An overview of the search and screening process is shown in Figure 1.

### Synthesis of Results

For each paper selected, we report descriptive results about the authors with the year of publication, the country of the first author, the types of technologies discussed, the role of AI in the technology, the type of study, and (if applicable) the methods and stakeholders involved for empirical data collection. Also, to provide an impression about practical approaches to responsible AI innovation in LTC, we report on responsible AI principles that the article addresses, and categorized papers in terms of their degree, level, and context of application. The *degree of application* means that we distinguish between papers that report on actual measures taken to address responsible AI in existing innovation practices, and papers that only contain recommendations to address RI at the level of the design and/or implementation of AI technologies. This distinction shows if responsible AI innovation is actually addressed in practice. With the *level of application*, we refer to classified papers as being related to a specific AI system, a particular category of AI technologies in LTC (e.g., care robots), or AI in LTC in general. This is relevant because it shows the context-specificity of the reported measures or recommendations. If applicable, we also report on the specific *context of application*, for instance a specific AI system, project, or geographical area in which responsible AI innovation is studied or practiced.

Our in-depth analysis of the included literature comprised an inductive thematic analysis to identify, analyze, and report repeated patterns across the articles (Braun & Clarke, 2006; Kiger & Varpio, 2020). The core concepts for our search and selection of papers provided starting points. Finally, this resulted in recurring focus areas in responsible AI innovation in LTC that emerge from the measures that are reported or recommended in the literature.

## Results

The systematic search in the digital libraries was conducted from June 2020 to September 2020. Figure 1 presents the flowchart for the selection of papers.

### Descriptive Results

Table 1 provides an overview of the key characteristics of the 25 papers included. Our systematic literature search yielded publications from the year 1974 up to 2021. All included papers were published since 2007 and the majority (*n* = 15) was published between 2018 and 2020. The identified papers mainly address RI in context of care robots (*n* = 12) and monitoring and smart home technology (*n* = 7). The papers differ in terms of how specific they describe the role of AI in their contexts. Nineteen of the included studies did not involve primary research but described the authors’ conceptual perspective on responsible AI innovation in LTC, the related technical approach, its feasibility, and/or an analysis of the literature. In total, six empirical studies were included, of which five used qualitative methods and one applied mixed methods.

**Table 1.** Descriptive Results

Publication	Country first author	Type of technology	Role of AI	Study type	Primary data collection
Anderson and Anderson (2011)	United States	Care robot	Determine when an overseer (e.g., a caregiver or family member) should be notified if the patient refuses to take the medication; engage in a natural language exchange; notify an overseer by e-mail when necessary	Technical approach	NA
Armbrust et al. (2011)	Germany	Autonomous robot	Serve as communication platform and roam around autonomously; take care of transportation and service needs of an older person	Technical approach	NA
Battistuzzi et al. (2018)	Italy	Socially assistive robots	Communicate through speech and gestures; provide health-related assistance, for example, remind users to take their medication; provide easy access to other technology	Conceptual perspective	NA
Chaaraoui et al. (2014)	Spain	Vision-based monitoring systems	Detection of home accidents; long-term behavior analysis; adapt privacy to the individual; balance information usefulness and the provided privacy to ensure that AAL services work properly	Technical approach	NA
Draper et al. (2014)	United Kingdom	Companion robot	Provide physical, cognitive, and social help in the home; co-learn with the householder; capable of empathic interaction	Qualitative	Focus groups with 55 older adults
Ferreira et al. (2019)	Portugal	Risk model (for AAL solutions)	Automatically learn from data about a user’s human, social, and technical context at that moment (e.g., time, location, previous interactions, type of connection/device, etc.) and decide what is the most transparent, secure, and usable way to both ask and retrieve the results of each request, to and from the application at hand	Technical approach	NA
Fiske et al. (2019)	Germany	Embodied AI applications	Perform high-level therapeutic interventions that used to be offered exclusively by highly trained, skilled health professionals such as psychotherapists, for example, responds independently of any expert human guidance to the client or patient through a virtually embodied presence, such as a robotic interface	Review and conceptual perspective	NA
Fong et al. (2012)	Australia	Smart home applications	Employ implicit sensing and complex reasoning mechanisms to try to understand the current context and situations of users and make appropriate adaptations according to users’ preferences	Technical approach	NA
Garner et al. (2016)	United Kingdom	Virtual carers	Administer preventative treatment (ensuring medicines are taken, providing dietary advice, managing exercise programs, etc.); aid in the event of a health/well-being problem	Qualitative	Focus groups with 13 older adults, among others
Hoque et al. (2009)	United States	Smart assisted home	Self-configuration, that is, the ability to integrate dynamically new software components and remove existing ones not needed any more	Technical approach	NA
Körtner (2016)	Austria	Social service robots	Patrol corridors, guide visitors to offices and accompany physiotherapy walking groups, play music, and stimulate participants suffering from dementia by means of sounds and a picture gallery	Conceptual perspective	NA
Langer et al. (2019)	Israel	Socially assistive robots	Provide assistance through social interactions; improve therapeutic compliance through verbal, noncontact, and personalized coaching	Review and conceptual perspective	NA
Mahoney et al. (2007)	United States	Smart homes	Proactively monitors and reports undesirable events	Review and conceptual perspective	NA
Matthias (2015)	United States	Care robots	Techniques like artificial neural networks, reinforcement learning, and genetic programming can be used so that the machine keeps learning and thus modifying its behavior in its deployment environment	Review and conceptual perspective	NA
Misselhorn (2020)	Germany	Artificial assistive systems	Autonomous artificial systems with moral capacities	Review and conceptual perspective	NA
Nguyen-Truong and Fritz (2018)	United States	Health-assistive smart home	Identify and predict health changes based on sensors that detect movements in the home, so that proactive interventions can be taken by family and/or nurses; learn to recognize motion patterns that are unique to the individual	Conceptual perspective	NA
Poulsen and Burmeister (2019)	Australia	Care robots	Replace one or multiple functions of a human caregiver	Qualitative	Heuristic evaluation with four experts and online survey with 102 global participants
Portacolone et al. (2020)	United States	Virtual artificial companions	Talk with users about topics that are meaningful to them. It is denoted that this application also relies on technicians “behind” the on-screen avatar who interact with users	Qualitative	Interviews, field notes and desk research
Takeda et al. (2019)	Japan	Support robot systems	Autonomous robots that use learning algorithms	Technical approach	NA
Tan and Taeihagh (2021)	Singapore	Robotics and autonomous systems	Engage in verbal and nonverbal interactions with older people; detect nerve signals on the arms and limbs and help to automatically move muscles (rehabilitation robots); check for vital signs to prevent the onset of diseases	Qualitative	25 interviews and document analysis
Thorstensen (2018)	Norway	Smart homes	Data are processed using machine learning with the aim to provide improved smart home functions that adapt automatically to preferences of individual residents	Conceptual perspective	NA
Vance et al. (2018)	Ireland	Risk model software	Interpret sensor data within the home as different events such as eating, sleeping, or watching TV and determine risk factors that allow health and social caregivers to put preventative measures in place to protect older adults from harm	Technical approach	NA
Wang et al. (2019)	United States	AI-assistive aids	Alert you or a friend about how you’re doing; predictive analytics; assess individual cognitive and physical status using traditional means and sensor technologies	Mixed methods	Focus groups and survey with 31 older adults
Yang et al. (2018)	China	Social robots	Detect embarrassing situations by a real-time object detection algorithm based on convolutional neural networks	Technical approach	NA
Yew (2020)	Singapore	Care robots	Reason, communicate, and learn from its perception of the surrounding environment, past experiences, and even errors; AI enables them to respond in socially interactive ways with humans	Review and conceptual perspective	NA

*Notes*: AI = artificial intelligence; AAL, ambient-assisted living; NA = not applicable.

The included studies indicated practical approaches to responsible AI innovation in LTC (see Table 2). Most papers report on responsible AI principles such as privacy, security, transparency, autonomy, trust, justice, and fairness (*n* = 22), while three papers discuss measures to address responsible AI innovation that are independent of principles (Misselhorn, 2020; Poulsen & Burmeister, 2019; Yew, 2020).

**Table 2.** Descriptive Results on Practical Approaches to Responsible AI Innovation

Publication	Responsible AI principles	Degree of application	Level of application	Context of application
Anderson and Anderson (2011)	Autonomy, nonmaleficence, beneficence	Actual measures	Specific	Instantiation of ETHEL (ETHical ELdercare system) in the Nao robot
Armbrust et al. (2011)	Privacy	Actual measures	Specific	Mobile robot Artos
Battistuzzi et al. (2018)	Autonomy, safety, well-being, among others	Solely recommendations	Specific	European-Japanese CARESSES project
Chaaraoui et al. (2014)	Privacy	Actual measures	Specific	vision@home project
Draper et al. (2014)	Autonomy, independence, enablement, safety, social connectedness	Solely recommendations	Specific	ACCOMPANY project, which uses the Care-O-bot 3 robot platform
Ferreira et al. (2019)	Trust, security	Solely recommendations	Category	Two use cases of the SoTRAACE prototype
Fiske et al. (2019)	Meaningful contact, equality, understandability, transparency, justice/nonbias, nonmaleficence	Solely recommendations	Category	NA
Fong et al. (2012)	Trust	Solely recommendations	Category	NA
Garner et al. (2016)	Multiple; transparency, autonomy, among others	Solely recommendations	Specific	RITA (Responsive Interactive Advocate) project
Hoque et al. (2009)	Trust, security	Solely recommendations	Category	NA
Körtner (2016)	Dignity, nonmaleficence, privacy	Actual measures	Specific	European STRANDS project
Langer et al. (2019)	Trust	Solely recommendations	Category	NA
Mahoney et al. (2007)	Autonomy, informed consent, beneficence, nonmaleficence, justice, fairness and equity, privacy	Solely recommendations	Category	NA
Matthias (2015)	Autonomy, trust	Solely recommendations	Category	NA
Misselhorn (2020)	Not centered around specific principles	Solely recommendations	General	NA
Nguyen-Truong and Fritz (2018)	Justice and fairness (diversity and nondiscrimination, accessibility), accuracy, privacy	Actual measures	Category	Portland metropolitan area
Poulsen and Burmeister (2019)	Not centered around specific principles, though principles such as safety and trust are mentioned	Solely recommendations	Category	NA
Portacolone et al. (2020)	Respect for persons, beneficence, nonmaleficence, justice	Solely recommendations	Category	Case study on http://www.care.coach, commercially available in the United States
Takeda et al. (2019)	Transparency, accountability	Actual measures	Specific	Physical human support robot
Tan and Taeihagh (2021)	Safety, privacy, accountability, autonomy and independence, social connectedness, dignity, justice	Actual measures	General	Singapore
Thorstensen (2018)	Privacy, transparency, safety, security, among others	Solely recommendations	Category	NA
Vance et al. (2018)	Transparency, accountability	Solely recommendations	Category	NA
Wang et al. (2019)	Transparency, privacy, and control	Solely recommendations	General	NA
Yang et al. (2018)	Privacy	Actual measures	Specific	A social robot was developed to demonstrate the performance of the algorithm for privacy situation detection
Yew (2020)	Not centered around specific principles, though some principles and frameworks are mentioned	Solely recommendations	Category	NA

*Notes*: AI = artificial intelligence; ACCOMPANY = Acceptable Robotic Companions for Ageing Years; CARRESSES = Culture-Aware Robots and Environmental Sensor Systems for Elderly Support; NA = not applicable; SoTRAACE = Socio-Technical Risk-Adaptable Access Control Model; STRANDS = Spatiotemporal Representations and Activities for Cognitive Control in Long-Term Scenarios.

#### Degree and level of application

Of the 25 papers, eight report on actual measures to address RI in existing AI innovation practices (see Table 2, *degree of application*). The other 17 papers solely provide recommendations for addressing RI in the design and implementation of AI technologies. While four of them discuss technical approaches and methods to address principles such as trust and transparency in AI, these were classified as “solely recommendations” because they do not report the respective methods being actually applied in existing AI technologies (Ferreira et al., 2019; Fong et al., 2012; Hoque et al., 2009; Vance et al., 2018).

Regarding the *level of application* (see Table 2), a distinction is made between papers that address responsible AI innovation at the level of a specific AI-based system (*n* = 9), in light of a particular category of AI-based technologies (*n =* 13), or without specific regard to particular types of technologies (*n* = 3).

It follows from the papers’ degree and level of application that six papers report on *actual measures* taken to address responsible AI innovation *at the level of specific AI-based systems* in LTC (Anderson & Anderson, 2011; Armbrust et al., 2011; Chaaraoui et al., 2014; Körtner, 2016; Takeda et al., 2019; Yang et al., 2018), four of which discuss approaches for the preservation of older adults’ privacy.

### Thematic Results

A thematic analysis was used to identify recurring main themes in the papers. Three overarching and interlinked themes were extracted that represent priorities in responsible AI innovation in LTC (see Table 3).

**Table 3.** Common Themes Reflected in the Papers

Publication	Common themes				
	1: User-oriented innovation			2: Framing AI as solution to RI issues	3: Context-sensitivity
	Users’ understanding and consent	Inclusivity and equity	Human dimension in AI-driven care		
Anderson and Anderson (2011)				x	x
Armbrust et al. (2011)			x	x	
Battistuzzi et al. (2018)					x
Chaaraoui et al. (2014)					x
Draper et al. (2014)	x			x	
Ferreira et al. (2019)	x			x	x
Fiske et al. (2019)		x	x		x
Fong et al. (2012)	x				
Garner et al. (2016)	x		x	x	x
Hoque et al. (2009)				x	
Körtner (2016)	x				x
Langer et al. (2019)	x		x		
Mahoney et al. (2007)	x	x			x
Matthias (2015)	x			x	
Misselhorn (2020)				x	x
Nguyen-Truong and Fritz (2018)	x	x	x		x
Poulsen and Burmeister (2019)			x	x	x
Portacolone et al. (2020)	x		x		
Takeda et al. (2019)	x				
Tan and Taeihagh (2021)	x	x	x		
Thorstensen (2018)	x		x		
Vance et al. (2018)	x		x		
Wang et al. (2019)	x				
Yang et al. (2018)				x	x
Yew (2020)		x	x	x	x

*Notes*: AI = artificial intelligence; RI = responsible innovation.

#### Theme 1: User-oriented AI innovation

In total, 19 papers provide recommendations or report on measures that are centered around the role of users, in particular older adults and their caregivers, in the design, and/or implementation of AI technologies. Three (interrelated) subthemes recur in the included papers (see Table 3). First, 15 papers provide recommendations relating to ***fostering users’ understanding and consent*** about the purposes of AI technologies, how to operate them, and how outcomes come about. For instance, Mahoney et al. (2007, p. 224) suggest to “avoid language that implies the technology does more than it actually does.” In addition, three papers provide suggestions regarding informing users about the purpose of AI technologies and their use of data. These include the provision of up-to-date printed information, and building feedback loops into the systems’ interfaces to help users understand how (their) data are used to predict health care needs (Körtner, 2016; Takeda et al., 2019; Wang et al., 2019). Five papers discuss that the variety and dynamics of users’ abilities to use, understand, or even consent to using the system must be accounted for in the design and/or implementation of AI technologies (Matthias, 2015; Takeda et al., 2019; Tan & Taeihagh, 2021; Thorstensen, 2018; Wang et al., 2019). For instance, Thorstensen (2018) suggests that privacy settings of smart home technologies can be constructed with a type of forward-looking consent based on users’ perspectives on, for example, privacy before their cognitive abilities decline. In addition, Matthias (2015) argues that care robots can better be equipped with user interfaces such as on-screen menus and buttons than with advanced AI-based natural language conversational interfaces, since the latter could deceive users about its capabilities and associated risks.

Second, five papers discuss the need to ***foster inclusivity and equity*** in the design and implementation of AI technologies. For instance, Nguyen-Truong and Fritz (2018) argue for better inclusion of minority populations and cultural differences in AI research and development to comply with the principles of fairness, diversity, and nondiscrimination. More specifically, they suggest that the eastern “interdependent” perspective on aging should be included by researchers and innovators when learning about desired functionalities and training AI systems. This, they argue, is because of the different ways of valuing privacy, parent–child relationships, connectivity, and outsourcing health and safety monitoring (in full) to technology, when compared to the Western “independent” perspective. In contrast, Yew (2020) stresses that macro-justice considerations such as equal care distribution may not necessarily need be taken into account during the design of care robots since their role is only to act in the best interests of specific individual users or user groups.

Third, 11 papers stress the importance of ***safeguarding the human dimension in AI-driven care***. This is firstly to foster social connectedness and avoid exacerbating the social isolation of older adults and secondly to have human supervision over AI-driven outcomes. One suggestion is that AI technologies should primarily be designed to *assist* human caregivers in supporting older adults, foster meaningful interactions between older adults, or substitute human caregivers when they are not available (Fiske et al., 2019; Garner et al., 2016; Portacolone et al., 2020; Yew, 2020). A contradictory recommendation is made by Armbrust et al. (2011), who argue that human involvement should be minimized during the use of a robotic system and that using AI could actually be a technical fix to privacy issues (also see Theme 2). More specifically, they suggest that human involvement is necessary when using a robotic system in older adults’ homes, but only during the final interpretation of a potential emergency situation, as this cannot (yet) be fully handled by state-of-the-art technology.

#### Theme 2: Framing AI as a solution to RI issues

In total, 11 papers discuss reasons and ways to use AI as a solution to RI issues (see Table 3). These papers actually position the use of AI as a *technical fix* to certain RI issues that are associated with supportive technologies in LTC, rather than as an RI problem in itself. The respective papers discuss conceptual, technical, or methodological approaches to *delegating some degree of responsibility* to AI technologies themselves. For instance, three papers discuss technical approaches to enabling AI technologies to determine what information should be shown to different users at a given moment (Chaaraoui et al., 2014; Ferreira et al., 2019; Yang et al., 2018). This is deemed important as it depends on the context of use and preferences of the individual older adult as to how much privacy-sensitive data can be made visible securely. Chaaraoui et al. (2014, p. 8910) state that “if the context is not correctly recognized by the intelligent monitoring services, then privacy protection will fail.” As discussed in the previous theme, it is deemed important that users can understand how AI technologies work. In this regard, two papers stress that AI technologies can themselves assess and evaluate users’ understanding to ensure that users do not overestimate the system’s abilities (Fiske et al., 2019; Matthias, 2015). Furthermore, four papers reflect on the need and possibilities to develop AI technologies with moral capacities; that is, capabilities to detect relevant ethical issues or principles and to deal with these issues or principles (Anderson & Anderson, 2011; Misselhorn, 2020; Poulsen & Burmeister, 2019; Yew, 2020). Misselhorn (2020) argues that at some point, human operators will be unable to fully control AI technologies due to their increasing levels of intelligence and autonomy. Therefore, it will supposedly become a necessity for AI technologies themselves to have moral capacities. Importantly, Yew (2020) stresses that such moral capacities should only be developed in strictly controlled laboratory conditions and that all users should ultimately stay in control over the operation.

#### Theme 3: Context-sensitivity

In total, 13 papers explicitly discuss the need and/or ways to be sensitive to the specific context of use of AI technologies in LTC when addressing RI. The included literature reflects this theme in multiple ways. First, some papers position context-sensitivity as a conditional factor for, or as an integral part of RI, regardless of particular issues at stake. For instance, four papers advocate a *hybrid approach* to responsible AI innovation as a means to achieving context-sensitivity in RI (Garner et al., 2016; Misselhorn, 2020; Poulsen & Burmeister, 2019; Yew, 2020). A hybrid approach to RI involves, on one hand, the *top-down* formulation of principles by experts and the realization of these principles in the generic design of AI technologies. On the other hand, it requires *bottom-up* engagement with the perspectives of individual users that are affected by AI technologies. In this way, the set of principles that guides AI’s behavior can be attuned to the specific context of use, but within the parameters of the general ethical framework (Misselhorn, 2020; Poulsen & Burmeister, 2019; Yew, 2020). Second, some papers provide information about particular contexts to which the respective insights on responsible AI innovation apply. For instance, Misselhorn (2020) points out that her methodological approach to implementing moral capacities in AI technologies, in which the care-dependent person decides which moral values are realized by the AI system, cannot be used in all LTC contexts. It is suggested that this particular approach is only applicable in care settings in which AI technologies are interacting with one user at a time and for users who are still able to make fundamental decisions regarding their own lives. Third, some papers discuss specific RI issues that require nuanced contextualization (Chaaraoui et al., 2014; Ferreira et al., 2019; Fiske et al., 2019; Körtner, 2016; Nguyen-Truong & Fritz, 2018; Yang et al., 2018). For instance, Fiske et al. (2019) argue that, depending on the available human resources in a care context, principally AI-driven care services are better than no care services at all.

## Discussion and Implications

While many studies recognize that responsible AI innovation in the LTC for older adults requires contextualization, limited studies address RI at the level of specific AI technologies and their local contexts of use. The ongoing scientific efforts to practice responsible AI innovation in LTC seem to be largely centered around the discussion of social and ethical concerns of AI, the perspectives of intended users and other stakeholders, and frameworks and principles that are adequate in this domain. We found limited empirical substantiation of practical measures that support responsible AI innovation and address principles in specific contexts of use.

Still, the reviewed literature does describe the rationales and ways to further address responsible AI innovation in LTC “in context.” Innovators often have difficulties in reconciling insights about user- or context-specific requirements or they even “decontextualized” design solutions because of their own need to offer somewhat standardized and scalable solutions (Peine, 2009; Peine & Moors, 2015). However, as Hagendorff (2020) argues, responsible AI innovation requires attention for specific technical systems and individual situations (also see Mittelstadt, 2019). Accordingly, even if the credibility of certain RI decisions in the design or implementation of AI technologies is high, their transferability to specific uses always requires contextualization. In this line, three papers identified in this review explicitly reflect on a *hybrid approach* to responsible AI innovation that involves top-down expert perspectives *and* bottom-up user perspectives. However, they do so as part of mulling over the delegation of moral responsibilities to AI (Misselhorn, 2020; Poulsen & Burmeister, 2019; Yew, 2020). This direction for RI approaches could be valuable, as technologies become more intelligent and autonomous and people—both designers and users with declining cognitive abilities—may no longer be able to take full “responsibility” for AI-based decisions and outcomes. At the same time, though, researchers and innovators should take into account a user-oriented perspective on AI innovation in LTC and continue to address user needs such as social connectedness, human supervision, and transparency.

In the meantime, it strikes us as pertinent that a hybrid approach to responsible AI innovation in LTC is pursued by human decision making involving older adults, their caregivers, and technology developers. This calls for innovators and future research about AI innovations in LTC to seek direction from principles and experts. Concurrently, innovators and researchers should continue to iteratively engage with users and people who are affected by specific AI technologies, even if some users such as people with dementia may have difficulties in expressing their feelings and wishes (Grigorovich et al., 2021; Suijkerbuijk et al., 2019). While user involvement in AI development and implementation may be important in any domain, this may especially be the case in the LTC for older adults, given the vulnerability of the target group.

### Implications for Research and Practice

Our findings have consequences for future frameworks for responsible AI innovation in LTC. The majority of included papers address the relevance and application of certain principles for responsible AI innovation, such as autonomy, informed consent, privacy, transparency, justice, fairness, and trust (see Table 2). However, given the limited empirical evidence of how principles are operationalized and applied in specific contexts of use, a fruitful direction for future research is to propose specific frameworks for responsible AI innovation in LTC. In line with the Responsible Research and Innovation perspective (Owen et al., 2013; Von Schomberg, 2013), such frameworks should clearly distinguish between RI *outcomes* and RI *processes.*

RI outcomes concern the characteristics that a given technology should possess and the societal needs or values and principles that must be addressed by innovation (Von Schomberg, 2013). RI processes are the actions, behavior, and activities that researchers and innovators undertake to support RI (Owen et al., 2013). As our results show, principles can be reflected in *RI outcomes*, for instance when personalized feedback loops in the system’s design foster users’ understanding and transparency (Takeda et al., 2019; Wang et al., 2019) and when forward-looking informed consent involves older adults’ perspectives on the technology’s use before their cognitive abilities decline (Thorstensen, 2018). Principles can also be reflected in *RI processes* such as inclusion of voices and data of minority populations to foster fairness, diversity, and nondiscrimination and ensure, for example, that technologies are made to fit both the eastern “interdependent” perspective on aging and the western “independent” perspective (Nguyen-Truong & Fritz, 2018). Future research could reveal how certain principles drive outcomes and processes of responsible AI innovation in LTC. Also, research could show how these RI outcomes and processes can be *flexibly attuned in context*, from early design to local use.

Another condition for such frameworks is that they are backed by illustrative empirical evidence that helps researchers and AI practitioners in LTC to flexibly address responsible AI innovation in different contexts of use. Further, such frameworks need to be continuously reshaped over time, since socially shared normative frameworks evolve with the emergence of new technologies and their routinization (Boenink et al., 2010; Kudina & Verbeek, 2019; Lehoux & Grimard, 2018). Lastly, it can be useful to learn from frameworks from other domains that may have moved the responsible development and deployment of AI technologies forward, like the six levels of driver-assistance technology that foster the safe integration of self-driving cars onto roadways (National Highway Traffic Safety Administration, n.d.; Topol, 2019).

In addition to the generation of frameworks, we call for expanding the empirical evidence on how responsible AI innovation is addressed in actual practice. It is important for researchers and innovators to explicate what decisions or actions in the design or implementation of AI technologies in LTC underpin RI, to think about local embedding and to more concrete suggestions at that level. To this respect, it could be useful to adopt the guidance ethics approach of Verbeek and Tijink (2020) or an agile approach for iteratively translating AI ethics guidelines to the specific context within which an AI system operates (Leijnen et al., 2020). Responsible AI innovation on the local level could directly contribute to the alignment of AI technologies and services with societal needs and values. This would reduce the risk of drawbacks such as low social adoption and unintended social and ethical consequences related to privacy, dignity, and autonomy, for instance. Without future research on the level of specific technologies and their local contexts of use, the scientific discourse on responsible AI innovation in LTC risks being largely confined to the hypothetical, devoid of the realities of real innovation practices and everyday life of innovators, older adults, caregivers, and other stakeholders of AI (Stahl & Coeckelbergh, 2016).

### Strengths and Limitations

This literature review included only papers that were fairly explicit about why the addressed technologies are labeled as “smart,” “intelligent,” or “adaptive,” for instance, and how AI plays a role in their operation. For this reason, discussions between the literature reviewers were held over a fair number of abstracts and full texts to reach consensus. In many cases it was decided to exclude specific papers because they insufficiently explicated whether AI was involved. Also, our review included academic research papers. Hence, it cannot claim to be complete and exhaustive in terms of the practical efforts that are or can be made to foster the responsible design and implementation of AI technologies in LTC. Incomplete access to the AI work being pursued by leading commercial technology companies is a limitation. A thorough examination of the gray literature could be useful to further reveal how this topic is addressed in practice. We acknowledge the challenge to be complete with regards to the dimensions of responsible AI innovation in LTC that can be addressed. Therefore, we have set up a comprehensive search strategy by using concepts from a global review on AI and ethics guidelines (Jobin et al., 2019), among others, that are expected to reasonably cover this theme. It is interesting for future studies to investigate more explicitly how RI is addressed in the context of AI technologies that facilitate decision making by clinicians in LTC. Through our focus on the LTC for older adults, our review may have missed out on relevant measures and strategies to address responsible AI innovation that emerge from a broader health care perspective or in other domains of health care. This review does not include papers that address AI technologies which are specifically targeted at the diagnosis and treatment of specific diseases common among older adults such as stroke, diabetes, chronic obstructive pulmonary disease, and cancer. To strengthen the insights from our review and foster cross-sectoral learning, future research could reveal how responsible AI innovation is practiced in other domains of health care.

## Conclusion

Based on our in-depth analysis of the relevant literature, we found three overarching themes that represent focus areas in practicing responsible AI innovation in the LTC for older adults: user-oriented AI innovation; framing AI as a solution to RI issues; and context-sensitivity. The results underpinning these themes provide insights into the efforts that can be made to foster the responsible design and implementation of AI technologies in LTC. This review therefore provides directions for AI researchers and practitioners when determining how AI technologies in LTC can be responsibly designed and implemented in the future. Importantly, a common thread in the studied literature is that responsible AI innovation requires a nuanced contextualization of RI issues and solutions. At the same time, the review points out that the current literature lacks clear substantiation about how certain measures affect responsible AI innovation in specific contexts. Future empirical research and frameworks on responsible AI innovation in LTC could reveal how certain principles are at the basis of RI *outcomes* and *processes*, from early design to local use. It could also be explored how these outcomes and processes can be *flexibly attuned in context*. Therefore, we recommend expanding the empirical evidence on RI at the level of specific AI technologies and their local contexts of use in LTC.

## Supplementary Material

gnab180_suppl_Supplementary_Material
