# Supplementary material for: Toward Responsible Artificial Intelligence in Long-Term Care: A Scoping Review on Practical Approaches
Source: Gerontologist. 2021 Dec 6;63(1):155–68. doi: 10.1093/geront/gnab180 (PMC9872770; doi:10.1093/geront/gnab180)
Supplement: gnab180_suppl_Supplementary_Material [file gnab180_suppl_supplementary_material.docx]

# Online Supplementary Material

**Supplementary Section A: PRISMA-ScR checklist**

| **Section/topic** | **#** | **Checklist item** | **Reported on page #** |
| --- | --- | --- | --- |
| **TITLE** | | | |
| Title | 1 | Identify the report as a scoping review. | 1 (Title page) |
| **ABSTRACT** | | | |
| Structured summary | 2 | Provide a structured summary that includes (as applicable) background, objectives, eligibility criteria, sources of evidence, charting methods, results, and conclusions that relate to the review questions and objectives. | 2 |
| **INTRODUCTION** | | | |
| Rationale | 3 | Describe the rationale for the review in the context of what is already known. Explain why the review questions/objectives lend themselves to a scoping review approach. | 3-5 |
| Objectives | 4 | Provide an explicit statement of the questions and objectives being addressed with reference to their key elements (e.g., population or participants, concepts, and context) or other relevant key elements used to conceptualize the review questions and/or objectives. | 3-5 |
| **METHODS** | | | |
| Protocol and registration | 5 | Indicate whether a review protocol exists; state if and where it can be accessed (e.g., a Web address); and if available, provide registration information, including the registration number. | N.A. |
| Eligibility criteria | 6 | Specify characteristics of the sources of evidence used as eligibility criteria (e.g., years considered, language, and publication status), and provide a rationale. | 5-6 |
| Information sources | 7 | Describe all information sources in the search (e.g., databases with dates of coverage and contact with authors to identify additional sources), as well as the date the most recent search was executed. | 5 |
| Search | 8 | Present the full electronic search strategy for at least 1 database, including any limits used, such that it could be repeated. | 5; Supplementary Section B |
| Study selection | 9 | State the process for selecting sources of evidence (i.e., screening and eligibility) included in the scoping review. | 6-7 |
| Data collection process | 10 | Describe the methods of charting data from the included sources of evidence (e.g., calibrated forms or forms that have been tested by the team before their use, and whether data charting was done independently or in duplicate) and any processes for obtaining and confirming data from investigators. | 7-8 |
| Data items | 11 | List and define all variables for which data were sought and any assumptions and simplifications made. | 7-8 |
| Risk of bias in individual studies | 12 | If done, provide a rationale for conducting a critical appraisal of included sources of evidence; describe the methods used and how this information was used in any data synthesis (if appropriate). | N.A. |
| Summary measures | 13 | Not applicable for scoping reviews. | N.A. |
| Synthesis of results | 14 | Describe the methods of handling and summarizing the data that were charted. | Qualitative analysis, pp. 7-8 |
| Risk of bias across studies | 15 | Not applicable for scoping reviews. | N.A. |
| Additional analyses | 16 | Not applicable for scoping reviews. | N.A. |
| **RESULTS** | | | |
| Selection of sources of evidence | 17 | Give numbers of sources of evidence screened, assessed for eligibility, and included in the review, with reasons for exclusions at each stage, ideally using a flow diagram. | 5-7, Figure 1 |
| Characteristics of sources of evidence | 18 | For each source of evidence, present characteristics for which data were charted and provide the citations. | 8-9, Table 1 and 2 |
| Critical appraisal within sources of evidence | 19 | If done, present data on critical appraisal of included sources of evidence (see item 12) | N.A. |
| Results of individual sources of evidence | 20 | For each included source of evidence, present the relevant data that were charted that relate to the review questions and objectives. | 8-12, Table 1-2 |
| Synthesis of results | 21 | Summarize and/or present the charting results as they relate to the review questions and objectives. | 9-12, Table 2-3 |
| Risk of bias across studies | 22 | Not applicable for scoping reviews. | N.A. |
| Additional analysis | 23 | Not applicable for scoping reviews. | N.A. |
| **DISCUSSION** | | | |
| Summary of evidence | 24 | Summarize the main results (including an overview of concepts, themes, and types of evidence available), link to the review questions and objectives, and consider the relevance to key groups. | 12-15 |
| Limitations | 25 | Discuss the limitations of the scoping review process. | 15-16 |
| Conclusions | 26 | Provide a general interpretation of the results with respect to the review questions and objectives, as well as potential implications and/or next steps. | 16 |
| **FUNDING** | | | |
| Funding | 27 | Describe sources of funding for the included sources of evidence, as well as sources of funding for the scoping review. Describe the role of the funders of the scoping review. | 17 |

Adapted from: Tricco, A. C., Lillie, E., Zarin, W., O’Brien, K. K., Colquhoun, H., Levac, D., Moher, D., Peters, M. D. J., Horsley, T., Weeks, L., Hempel, S., Akl, E. A., Chang, C., McGowan, J., Stewart, L., Hartling, L., Aldcroft, A., Wilson, M. G., Garritty, C., … Straus, S. E. (2018). PRISMA extension for scoping reviews (PRISMA-ScR): Checklist and explanation. In Annals of Internal Medicine (Vol. 169, Issue 7, pp. 467–473). American College of Physicians. https://doi.org/10.7326/M18-0850

**Supplementary Section B: Search terms and query**

We conducted a systematic literature search in five electronic databases (PubMed, Web of Science, Scopus, PsycInfo, CINAHL) using a comprehensive search string that covers a wide set of terms related to the three core search concepts: long-term care, Artificial Intelligence, Responsible Innovation. Below, the search string is shown per database.

**Search strategy for PubMed (17 June 2020)**

| **Search** | **Query** | **Results** |
| --- | --- | --- |
| **#12** | **(#4 AND #10 AND #11)** | **1,014** |
| **#11** | **"Ethics"[Mesh] OR "Privacy"[Mesh] OR "Freedom"[Mesh] OR "Personal Autonomy"[Mesh] OR responsib*[tiab] OR ethic*[tiab] OR trustworth*[tiab] OR trust[tiab] OR trusting[tiab] OR moral*[tiab] OR bioethic*[tiab] OR metaethic*[tiab] OR sustainab*[tiab] OR desirab*[tiab] OR anticipat*[tiab] OR reflecti*[tiab] OR deliberat*[tiab] OR responsive*[tiab] OR transparen*[tiab] OR justice[tiab] OR common good[tiab] OR justifiab*[tiab] OR fairness[tiab] OR fair[tiab] OR non-maleficen*[tiab] OR nonmaleficen*[tiab] OR privacy[tiab] OR beneficen*[tiab] OR freedom*[tiab] OR autonom*[tiab] OR self determination*[tiab] OR free will*[tiab] OR dignit*[tiab] OR solidarit*[tiab]** | **1,679,762** |
| **#10** | **(#6 OR #8 OR #9)** | **527,260** |
| **#9** | **"Artificial Intelligence"[Mesh] OR artificial intelligen*[tiab] OR algorithm*[tiab] OR computational intelligen*[tiab] OR machine intelligen*[tiab] OR computer reasoning*[tiab] OR ai[tiab] OR rule based[tiab] OR machine learning*[tiab] OR deep learning*[tiab] OR hierarchical learning*[tiab] OR computer heuristic*[tiab] OR expert system*[tiab] OR supervised learning*[tiab] OR unsupervised learning*[tiab] OR reinforcement learning*[tiab] OR natural language processing*[tiab] OR computer vision*[tiab] OR machine vision*[tiab] OR image recognition*[tiab] OR speech recognition*[tiab] OR neural network*[tiab] OR perceptron*[tiab] OR connectionist model*[tiab]** | **409,684** |
| **#8** | **(#2 AND #7)** | **42,422** |
| **#7** | **((monitoring*[tiab] OR surveillanc*[tiab] OR sensing[tiab] OR sensoring[tiab] OR sensor[tiab] OR sensors[tiab] OR sensoric*[tiab] OR sensory[tiab]) AND (system[tiab] OR systems[tiab] OR technolog*[tiab] OR software*[tiab] OR tool[tiab] OR tools[tiab] OR application*[tiab]))** | **355,587** |
| **#6** | **(#2 AND #5)** | **95,141** |
| **#5** | **robot*[tiab] OR telerobot*[tiab] OR “Decision Support Systems, Clinical"[Mesh] OR "Decision Support Techniques"[Mesh] OR decision support*[tiab] OR decision aid*[tiab] OR smart home*[tiab] OR assistive technolog*[tiab] OR ambient technolog*[tiab] OR active[tiab] OR assisted living[tiab] OR active assisted living[tiab] OR ambient assisted living[tiab] OR gaming[tiab] OR game[tiab] OR games[tiab] OR virtual companion*[tiab] OR virtual realit*[tiab] OR augmented realit*[tiab] OR mixed realit*[tiab]** | **1,131,059** |
| **#4** | **(#1 OR (#2 AND #3))** | **414,924** |
| **#3** | **gerontechnolog*[tiab] OR gerotechnology*[tiab] OR “age tech”[tiab] OR agetech[tiab]** | **109** |
| **#2** | **smart[tiab] OR intelligent[tiab] OR adaptive[tiab] OR predict*[tiab] OR autonomous*[tiab] OR self learning*[tiab] OR data driven*[tiab]** | **1,780,992** |
| **#1** | **"Homes for the Aged"[Mesh] OR "Health Services for the Aged"[Mesh] OR home of the aged[tiab] OR homes for the aged[tiab] OR old age home*[tiab] OR healthy aging[tiab] OR active aging[tiab] OR healthy ageing[tiab] OR active ageing[tiab] OR silver econom*[tiab] OR (("Aged"[Mesh] OR "Aged, 80 and over"[Mesh] OR "Frail Elderly"[Mesh] OR "Geriatrics"[Mesh] OR "Geriatric Psychiatry"[Mesh] OR "Geriatric Nursing"[Mesh] OR elder*[tiab] OR eldest[tiab] OR geriatri*[tiab] OR gerontol*[tiab] OR old age*[tiab] OR oldest old*[tiab] OR senior*[tiab] OR senium[tiab] OR very old*[tiab] OR septuagenarian*[tiab] OR octagenarian*[tiab] OR octogenarian*[tiab] OR nonagenarian*[tiab] OR centarian*[tiab] OR centenarian*[tiab] OR supercentenarian*[tiab] OR older people[tiab] OR older subject*[tiab] OR older patient*[tiab] OR older age*[tiab] OR older adult*[tiab] OR older man[tiab] OR older men[tiab] OR older male*[tiab] OR older woman[tiab] OR older women[tiab] OR older female*[tiab] OR older population*[tiab] OR older person*[tiab] OR ageing*[tiab] OR aging*[tiab] OR "Dementia"[Mesh] OR dementi*[tiab] OR amenti* OR alzheimer*[tiab] OR boomer*[tiab] OR later life*[tiab]) AND ("Residential Facilities"[Mesh] OR "Rehabilitation Centers"[Mesh:NoExp] OR "Independent Living"[Mesh] OR care[tiab] OR assisted liv*[tiab] OR rehabilitation[tiab] OR residential*[tiab] OR community-dwelling*[tiab] OR home-dwelling*[tiab] OR independent living*[tiab] OR nursing home*[tiab]))** | **414,911** |

**Search strategy for Clarivate Analytics/Web of Science Core Collection (14 July 2020)**

| **Set** | Query | **Results** |
| --- | --- | --- |
| **#10** | #8 AND #9 AND #7 | **807** |
| **#9** | (#2 AND #3) OR (#2 AND #4) OR #5 | **1,737,274** |
| **#8** | #1 OR (#2 AND #6) | **182,237** |
| **#7** | TOPIC: (“responsib*” OR “ethic*” OR “trustworth*” OR “trust” OR “trusting” OR “moral*” OR “bioethic*” OR “metaethic*” OR “sustainab*” OR “desirab*” OR “anticipat*” OR “reflecti*” OR “deliberat*” OR “responsive*” OR “transparen*” OR “justice” OR “common good” OR “justifiab*” OR “fairness” OR “fair” OR “non-maleficen*” OR “nonmaleficen*” OR “privacy” OR “beneficen*” OR “freedom*” OR “autonom*” OR “self determination*” OR “free will*” OR “dignit*” OR “solidarit*”) | **3,220,694** |
| **#6** | TOPIC: (“gerontechnolog*” OR “gerotechnology*” OR “age tech” OR “agetech”) | **171** |
| **#5** | TOPIC: (“artificial intelligen*” OR “algorithm*” OR “computational intelligen*” OR “machine intelligen*” OR “computer reasoning*” OR “ai” OR “rule based” OR “machine learning*” OR “deep learning*” OR “hierarchical learning*” OR “computer heuristic*” OR “expert system*” OR “supervised learning*” OR “unsupervised learning*” OR “reinforcement learning*” OR “natural language processing*” OR “computer vision*” OR “machine vision*” OR “image recognition*” OR “speech recognition*” OR “neural network*” OR “perceptron*” OR “connectionist model*”) | **1,499,927** |
| **#4** | TOPIC: (((“monitoring*” OR “surveillanc*” OR “sensing” OR “sensoring” OR “sensor” OR “sensors” OR “sensoric*” OR “sensory”) AND (“system” OR “systems” OR “technolog*” OR “software*” OR “tool” OR “tools” OR “application*”))) | **782,021** |
| **#3** | TOPIC: (“robot*” OR “telerobot*” OR “decision support*” OR “decision aid*” OR “smart home*” OR “assistive technolog*” OR “ambient technolog*” OR “active” OR “assisted living” OR “active assisted living” OR “ambient assisted living” OR “gaming” OR “game” OR “games” OR “virtual companion*” OR “virtual realit*” OR “augmented realit*” OR “mixed realit*”) | **1,845,046** |
| **#2** | TOPIC: (“smart” OR “intelligent” OR “adaptive” OR “predict*” OR “autonomous*” OR “self learning*” OR “data driven*”) | **3,613,678** |
| **#1** | TOPIC: (“home of the aged” OR “homes for the aged” OR “old age home*” OR “healthy aging” OR “active aging” OR “healthy ageing” OR “active ageing” OR “silver econom*” OR ((“elder*” OR “eldest” OR “geriatri*” OR “gerontol*” OR “old age*” OR “oldest old*” OR “senior*” OR “senium” OR “very old*” OR “septuagenarian*” OR “octagenarian*” OR “octogenarian*” OR “nonagenarian*” OR “centarian*” OR “centenarian*” OR “supercentenarian*” OR “older people” OR “older subject*” OR “older patient*” OR “older age*” OR “older adult*” OR “older man” OR “older men” OR “older male*” OR “older woman” OR “older women” OR “older female*” OR “older population*” OR “older person*” OR “ageing*” OR “aging*” OR “amenti*” OR “alzheimer*” OR “boomer*” OR “later life*”) AND (“care” OR “assisted liv*” OR “rehabilitation” OR “residential*” OR “community-dwelling*” OR “home-dwelling*” OR “independent living*” OR “nursing home*”))) | **182,224** |

**Search strategy for Elsevier/Scopus (14 July 2020)**

| **History**  **Count** | **Search Terms** | **Results** |
| --- | --- | --- |
| **#10** | #7 AND #8 AND #9 | **2,469** |
| **#9** | (#2 AND #3) OR (#2 AND #4) OR #5 | **4,470,340** |
| **#8** | #1 OR (#2 AND #6) | **431,508** |
| **#7** | TITLE-ABS-KEY ("responsib*" OR "ethic*" OR "trustworth*" OR "trust" OR "trusting" OR "moral*" OR "bioethic*" OR "metaethic*" OR "sustainab*" OR "desirab*" OR "anticipat*" OR "reflecti*" OR "deliberat*" OR "responsive*" OR "transparen*" OR "justice" OR "common good" OR "justifiab*" OR "fairness" OR "fair" OR "non-maleficen*" OR "nonmaleficen*" OR "privacy" OR "beneficen*" OR "freedom*" OR "autonom*" OR "self determination*" OR "free will*" OR "dignit*" OR "solidarit*") | **5,414,117** |
| **#6** | TITLE-ABS-KEY ("gerontechnolog*" OR "gerotechnology*" OR "age tech" OR "agetech") | **556** |
| **#5** | TITLE-ABS-KEY ("artificial intelligen*" OR "algorithm*" OR "computational intelligen*" OR "machine intelligen*" OR "computer reasoning*" OR "ai" OR "rule based" OR "machine learning*" OR "deep learning*" OR "hierarchical learning*" OR "computer heuristic*" OR "expert system*" OR "supervised learning*" OR "unsupervised learning*" OR "reinforcement learning*" OR "natural language processing*" OR "computer vision*" OR "machine vision*" OR "image recognition*" OR "speech recognition*" OR "neural network*" OR "perceptron*" OR "connectionist model*") | **3,947,888** |
| **#4** | TITLE-ABS-KEY ((("monitoring*" OR "surveillanc*" OR "sensing" OR "sensoring" OR "sensor" OR "sensors" OR "sensoric*" OR "sensory") AND ("system" OR "systems" OR "technolog*" OR "software*" OR "tool" OR "tools" OR "application*"))) | **1,993,124** |
| **#3** | TITLE-ABS-KEY ("robot*" OR "telerobot*" OR "decision support*" OR "decision aid*" OR "smart home*" OR "assistive technolog*" OR "ambient technolog*" OR "active" OR "assisted living" OR "active assisted living" OR "ambient assisted living" OR "gaming" OR "game" OR "games" OR "virtual companion*" OR "virtual realit*" OR "augmented realit*" OR "mixed realit*") | **3,284,846** |
| **#2** | TITLE-ABS-KEY ("smart" OR "intelligent" OR "adaptive" OR "predict*" OR "autonomous*" OR "self learning*" OR "data driven*") | **5,783,232** |
| **#1** | TITLE-ABS-KEY ("home of the aged" OR "homes for the aged" OR "old age home*" OR "healthy aging" OR "active aging" OR "healthy ageing" OR "active ageing" OR "silver econom*" OR (("elder*" OR "eldest" OR "geriatri*" OR "gerontol*" OR "old age*" OR "oldest old*" OR "senior*" OR "senium" OR "very old*" OR "septuagenarian*" OR "octagenarian*" OR "octogenarian*" OR "nonagenarian*" OR "centarian*" OR "centenarian*" OR "supercentenarian*" OR "older people" OR "older subject*" OR "older patient*" OR "older age*" OR "older adult*" OR "older man" OR "older men" OR "older male*" OR "older woman" OR "older women" OR "older female*" OR "older population*" OR "older person*" OR "ageing*" OR "aging*" OR "amenti*" OR "alzheimer*" OR "boomer*" OR "later life*") AND ("care" OR "assisted liv*" OR "rehabilitation" OR "residential*" OR "community-dwelling*" OR "home-dwelling*" OR "independent living*" OR "nursing home*"))) | **431,467** |

**Search strategy for Ebsco/APA PsycINFO (21 August 2020)**

| **#** | **Query** | **Results** |
| --- | --- | --- |
| **S10** | S8 AND S9 AND S7 | **192** |
| **S9** | (S2 AND S3) OR (S2 AND S4) OR S5 | **118,077** |
| **S8** | S1 OR (S2 AND S6) | **63,037** |
| **S7** | DE "Ethics" OR DE "Bioethics" OR DE "Business Ethics" OR DE "Consumer Ethics" OR DE "Experimental Ethics" OR DE "Professional Ethics" OR DE "Professional Ethics" OR DE "Boundary Crossings" OR DE "Boundary Violations" OR DE "Conflict of Interest" OR DE "Dual Relationships" OR DE "Duty to Protect" OR DE "Duty to Warn" OR DE "Professional Standards" OR DE "Trust (Social Behavior)" OR DE "Morality" OR DE "Research Transparency" OR DE "Fairness" OR DE "Justice" OR DE "Privacy" OR DE "Autonomy" OR DE "Freedom" OR DE "Self-Determination" OR DE "Volition" OR DE "Dignity" OR DE "Solidarity" OR TI("responsib*" OR "ethic*" OR "trustworth*" OR "trust" OR "trusting" OR "moral*" OR "bioethic*" OR "metaethic*" OR "sustainab*" OR "desirab*" OR "anticipat*" OR "reflecti*" OR "deliberat*" OR "responsive*" OR "transparen*" OR "justice" OR "common good" OR "justifiab*" OR "fairness" OR "fair" OR "non-maleficen*" OR "nonmaleficen*" OR "privacy" OR "beneficen*" OR "freedom*" OR "autonom*" OR "self determination*" OR "free will*" OR "dignit*" OR "solidarit*") OR AB("responsib*" OR "ethic*" OR "trustworth*" OR "trust" OR "trusting" OR "moral*" OR "bioethic*" OR "metaethic*" OR "sustainab*" OR "desirab*" OR "anticipat*" OR "reflecti*" OR "deliberat*" OR "responsive*" OR "transparen*" OR "justice" OR "common good" OR "justifiab*" OR "fairness" OR "fair" OR "non-maleficen*" OR "nonmaleficen*" OR "privacy" OR "beneficen*" OR "freedom*" OR "autonom*" OR "self determination*" OR "free will*" OR "dignit*" OR "solidarit*") OR KW("responsib*" OR "ethic*" OR "trustworth*" OR "trust" OR "trusting" OR "moral*" OR "bioethic*" OR "metaethic*" OR "sustainab*" OR "desirab*" OR "anticipat*" OR "reflecti*" OR "deliberat*" OR "responsive*" OR "transparen*" OR "justice" OR "common good" OR "justifiab*" OR "fairness" OR "fair" OR "non-maleficen*" OR "nonmaleficen*" OR "privacy" OR "beneficen*" OR "freedom*" OR "autonom*" OR "self determination*" OR "free will*" OR "dignit*" OR "solidarit*") | **633,724** |
| **S6** | TI("gerontechnolog*" OR "gerotechnology*" OR "age tech" OR "agetech") OR AB("gerontechnolog*" OR "gerotechnology*" OR "age tech" OR "agetech") OR KW("gerontechnolog*" OR "gerotechnology*" OR "age tech" OR "agetech") | **155** |
| **S5** | DE "Artificial Intelligence" OR DE "Machine Learning" OR DE "Natural Language Processing" OR DE "Machine Learning Algorithms" OR DE "Extreme Learning Machine" OR DE "Unsupervised Learning" OR DE "Heuristic Modeling" OR DE "Expert Systems" OR DE "Automated Speech Recognition" OR DE "Artificial Neural Networks" OR DE "Computer Simulation" OR DE "Neural Networks" OR TI("artificial intelligen*" OR "algorithm*" OR "computational intelligen*" OR "machine intelligen*" OR "computer reasoning*" OR "ai" OR "rule based" OR "machine learning*" OR "deep learning*" OR "hierarchical learning*" OR "computer heuristic*" OR "expert system*" OR "supervised learning*" OR "unsupervised learning*" OR "reinforcement learning*" OR "natural language processing*" OR "computer vision*" OR "machine vision*" OR "image recognition*" OR "speech recognition*" OR "neural network*" OR "perceptron*" OR "connectionist model*") OR AB("artificial intelligen*" OR "algorithm*" OR "computational intelligen*" OR "machine intelligen*" OR "computer reasoning*" OR "ai" OR "rule based" OR "machine learning*" OR "deep learning*" OR "hierarchical learning*" OR "computer heuristic*" OR "expert system*" OR "supervised learning*" OR "unsupervised learning*" OR "reinforcement learning*" OR "natural language processing*" OR "computer vision*" OR "machine vision*" OR "image recognition*" OR "speech recognition*" OR "neural network*" OR "perceptron*" OR "connectionist model*") OR KW("artificial intelligen*" OR "algorithm*" OR "computational intelligen*" OR "machine intelligen*" OR "computer reasoning*" OR "ai" OR "rule based" OR "machine learning*" OR "deep learning*" OR "hierarchical learning*" OR "computer heuristic*" OR "expert system*" OR "supervised learning*" OR "unsupervised learning*" OR "reinforcement learning*" OR "natural language processing*" OR "computer vision*" OR "machine vision*" OR "image recognition*" OR "speech recognition*" OR "neural network*" OR "perceptron*" OR "connectionist model*") | **91,267** |
| **S4** | TI((("monitoring*" OR "surveillanc*" OR "sensing" OR "sensoring" OR "sensor" OR "sensors" OR "sensoric*" OR "sensory") AND ("system" OR "systems" OR "technolog*" OR "software*" OR "tool" OR "tools" OR "application*"))) OR AB((("monitoring*" OR "surveillanc*" OR "sensing" OR "sensoring" OR "sensor" OR "sensors" OR "sensoric*" OR "sensory") AND ("system" OR "systems" OR "technolog*" OR "software*" OR "tool" OR "tools" OR "application*"))) OR KW((("monitoring*" OR "surveillanc*" OR "sensing" OR "sensoring" OR "sensor" OR "sensors" OR "sensoric*" OR "sensory") AND ("system" OR "systems" OR "technolog*" OR "software*" OR "tool" OR "tools" OR "application*"))) | **44,314** |
| **S3** | DE "Robotics" OR DE "Avatars" OR DE "Human Robot Interaction" OR DE "Intelligent Agents" OR DE "Conversational Agents" OR DE "Social Robotics" OR DE "Decision Support Systems") OR DE "Assistive Technology" OR DE "Assisted Living" OR DE "Computer Games" OR DE "Virtual Reality" OR DE "Augmented Reality" OR TI("robot*" OR "telerobot*" OR "decision support*" OR "decision aid*" OR "smart home*" OR "assistive technolog*" OR "ambient technolog*" OR "active" OR "assisted living" OR "active assisted living" OR "ambient assisted living" OR "gaming" OR "game" OR "games" OR "virtual companion*" OR "virtual realit*" OR "augmented realit*" OR "mixed realit*") OR AB("robot*" OR "telerobot*" OR "decision support*" OR "decision aid*" OR "smart home*" OR "assistive technolog*" OR "ambient technolog*" OR "active" OR "assisted living" OR "active assisted living" OR "ambient assisted living" OR "gaming" OR "game" OR "games" OR "virtual companion*" OR "virtual realit*" OR "augmented realit*" OR "mixed realit*") OR KW("robot*" OR "telerobot*" OR "decision support*" OR "decision aid*" OR "smart home*" OR "assistive technolog*" OR "ambient technolog*" OR "active" OR "assisted living" OR "active assisted living" OR "ambient assisted living" OR "gaming" OR "game" OR "games" OR "virtual companion*" OR "virtual realit*" OR "augmented realit*" OR "mixed realit*") | **186,530** |
| **S2** | TI("smart" OR "intelligent" OR "adaptive" OR "predict*" OR "autonomous*" OR "self learning*" OR "data driven*") OR AB("smart" OR "intelligent" OR "adaptive" OR "predict*" OR "autonomous*" OR "self learning*" OR "data driven*") OR KW("smart" OR "intelligent" OR "adaptive" OR "predict*" OR "autonomous*" OR "self learning*" OR "data driven*") | **529,476** |
| **S1** | DE "Nursing Homes" OR DE "Elder Care" OR DE "Nursing Home Residents" OR TI("home of the aged" OR "homes for the aged" OR "old age home*" OR "healthy aging" OR "active aging" OR "healthy ageing" OR "active ageing" OR "silver econom*" OR (("elder*" OR "eldest" OR "geriatri*" OR "gerontol*" OR "old age*" OR "oldest old*" OR "senior*" OR "senium" OR "very old*" OR "septuagenarian*" OR "octagenarian*" OR "octogenarian*" OR "nonagenarian*" OR "centarian*" OR "centenarian*" OR "supercentenarian*" OR "older people" OR "older subject*" OR "older patient*" OR "older age*" OR "older adult*" OR "older man" OR "older men" OR "older male*" OR "older woman" OR "older women" OR "older female*" OR "older population*" OR "older person*" OR "ageing*" OR "aging*" OR "amenti*" OR "alzheimer*" OR "boomer*" OR "later life*") AND ("care" OR "assisted liv*" OR "rehabilitation" OR "residential*" OR "community-dwelling*" OR "home-dwelling*" OR "independent living*" OR "nursing home*"))) OR AB("home of the aged" OR "homes for the aged" OR "old age home*" OR "healthy aging" OR "active aging" OR "healthy ageing" OR "active ageing" OR "silver econom*" OR (("elder*" OR "eldest" OR "geriatri*" OR "gerontol*" OR "old age*" OR "oldest old*" OR "senior*" OR "senium" OR "very old*" OR "septuagenarian*" OR "octagenarian*" OR "octogenarian*" OR "nonagenarian*" OR "centarian*" OR "centenarian*" OR "supercentenarian*" OR "older people" OR "older subject*" OR "older patient*" OR "older age*" OR "older adult*" OR "older man" OR "older men" OR "older male*" OR "older woman" OR "older women" OR "older female*" OR "older population*" OR "older person*" OR "ageing*" OR "aging*" OR "amenti*" OR "alzheimer*" OR "boomer*" OR "later life*") AND ("care" OR "assisted liv*" OR "rehabilitation" OR "residential*" OR "community-dwelling*" OR "home-dwelling*" OR "independent living*" OR "nursing home*"))) OR KW("home of the aged" OR "homes for the aged" OR "old age home*" OR "healthy aging" OR "active aging" OR "healthy ageing" OR "active ageing" OR "silver econom*" OR (("elder*" OR "eldest" OR "geriatri*" OR "gerontol*" OR "old age*" OR "oldest old*" OR "senior*" OR "senium" OR "very old*" OR "septuagenarian*" OR "octagenarian*" OR "octogenarian*" OR "nonagenarian*" OR "centarian*" OR "centenarian*" OR "supercentenarian*" OR "older people" OR "older subject*" OR "older patient*" OR "older age*" OR "older adult*" OR "older man" OR "older men" OR "older male*" OR "older woman" OR "older women" OR "older female*" OR "older population*" OR "older person*" OR "ageing*" OR "aging*" OR "amenti*" OR "alzheimer*" OR "boomer*" OR "later life*") AND ("care" OR "assisted liv*" OR "rehabilitation" OR "residential*" OR "community-dwelling*" OR "home-dwelling*" OR "independent living*" OR "nursing home*"))) | **63,025** |

**Search strategy for Ebsco/CINAHL (8 September 2020)**

| **#** | **Query** | **Results** |
| --- | --- | --- |
| **S10** | S8 AND S9 AND S7 | **309** |
| **S9** | (S2 AND S3) OR (S2 AND S4) OR S5 | **98,380** |
| **S8** | S1 OR (S6 AND S2) | **130,268** |
| **S7** | MH "Privacy and Confidentiality+" OR MH "Ethics+" OR MH "Morale" OR MH "Morals+" OR MH "Beneficence" OR MH "Bioethics" OR MH "Decision Making, Ethical" OR MH "Conflict of Interest" OR MH "Ethics, Professional+" OR MH "Personal Boundaries+" OR MH "Trust" OR MH "Autonomy+" OR MH "Freedom" OR MH "Human Dignity" OR MH "Volition" OR TI("responsib*" OR "ethic*" OR "trustworth*" OR "trust" OR "trusting" OR "moral*" OR "bioethic*" OR "metaethic*" OR "sustainab*" OR "desirab*" OR "anticipat*" OR "reflecti*" OR "deliberat*" OR "responsive*" OR "transparen*" OR "justice" OR "common good" OR "justifiab*" OR "fairness" OR "fair" OR "non-maleficen*" OR "nonmaleficen*" OR "privacy" OR "beneficen*" OR "freedom*" OR "autonom*" OR "self determination*" OR "free will*" OR "dignit*" OR "solidarit*") OR AB("responsib*" OR "ethic*" OR "trustworth*" OR "trust" OR "trusting" OR "moral*" OR "bioethic*" OR "metaethic*" OR "sustainab*" OR "desirab*" OR "anticipat*" OR "reflecti*" OR "deliberat*" OR "responsive*" OR "transparen*" OR "justice" OR "common good" OR "justifiab*" OR "fairness" OR "fair" OR "non-maleficen*" OR "nonmaleficen*" OR "privacy" OR "beneficen*" OR "freedom*" OR "autonom*" OR "self determination*" OR "free will*" OR "dignit*" OR "solidarit*") OR KW("responsib*" OR "ethic*" OR "trustworth*" OR "trust" OR "trusting" OR "moral*" OR "bioethic*" OR "metaethic*" OR "sustainab*" OR "desirab*" OR "anticipat*" OR "reflecti*" OR "deliberat*" OR "responsive*" OR "transparen*" OR "justice" OR "common good" OR "justifiab*" OR "fairness" OR "fair" OR "non-maleficen*" OR "nonmaleficen*" OR "privacy" OR "beneficen*" OR "freedom*" OR "autonom*" OR "self determination*" OR "free will*" OR "dignit*" OR "solidarit*") | **471,397** |
| **S6** | TI("gerontechnolog*" OR "gerotechnology*" OR "age tech" OR "agetech") OR AB("gerontechnolog*" OR "gerotechnology*" OR "age tech" OR "agetech") OR KW("gerontechnolog*" OR "gerotechnology*" OR "age tech" OR "agetech") | **125** |
| **S5** | MH "Artificial Intelligence+" OR MH "Machine Learning+" OR MH "Natural Language Processing" OR MH "Neural Networks (Computer)" OR MH "Computer Simulation" OR TI("artificial intelligen*" OR "algorithm*" OR "computational intelligen*" OR "machine intelligen*" OR "computer reasoning*" OR "ai" OR "rule based" OR "machine learning*" OR "deep learning*" OR "hierarchical learning*" OR "computer heuristic*" OR "expert system*" OR "supervised learning*" OR "unsupervised learning*" OR "reinforcement learning*" OR "natural language processing*" OR "computer vision*" OR "machine vision*" OR "image recognition*" OR "speech recognition*" OR "neural network*" OR "perceptron*" OR "connectionist model*") OR AB("artificial intelligen*" OR "algorithm*" OR "computational intelligen*" OR "machine intelligen*" OR "computer reasoning*" OR "ai" OR "rule based" OR "machine learning*" OR "deep learning*" OR "hierarchical learning*" OR "computer heuristic*" OR "expert system*" OR "supervised learning*" OR "unsupervised learning*" OR "reinforcement learning*" OR "natural language processing*" OR "computer vision*" OR "machine vision*" OR "image recognition*" OR "speech recognition*" OR "neural network*" OR "perceptron*" OR "connectionist model*") OR KW("artificial intelligen*" OR "algorithm*" OR "computational intelligen*" OR "machine intelligen*" OR "computer reasoning*" OR "ai" OR "rule based" OR "machine learning*" OR "deep learning*" OR "hierarchical learning*" OR "computer heuristic*" OR "expert system*" OR "supervised learning*" OR "unsupervised learning*" OR "reinforcement learning*" OR "natural language processing*" OR "computer vision*" OR "machine vision*" OR "image recognition*" OR "speech recognition*" OR "neural network*" OR "perceptron*" OR "connectionist model*") | **79,695** |
| **S4** | MH "Wearable Sensors" OR TI((("monitoring*" OR "surveillanc*" OR "sensing" OR "sensoring" OR "sensor" OR "sensors" OR "sensoric*" OR "sensory") AND ("system" OR "systems" OR "technolog*" OR "software*" OR "tool" OR "tools" OR "application*"))) OR AB((("monitoring*" OR "surveillanc*" OR "sensing" OR "sensoring" OR "sensor" OR "sensors" OR "sensoric*" OR "sensory") AND ("system" OR "systems" OR "technolog*" OR "software*" OR "tool" OR "tools" OR "application*"))) OR KW((("monitoring*" OR "surveillanc*" OR "sensing" OR "sensoring" OR "sensor" OR "sensors" OR "sensoric*" OR "sensory") AND ("system" OR "systems" OR "technolog*" OR "software*" OR "tool" OR "tools" OR "application*"))) | **54,168** |
| **S3** | MH "Robotics" OR MH "Decision Support Systems, Clinical" OR MH "Decision Support Systems, Management" OR MH "Assistive Technology" OR MH "Assisted Living" OR MH "Virtual Reality" OR MH "Augmented Reality" OR TI("robot*" OR "telerobot*" OR "decision support*" OR "decision aid*" OR "smart home*" OR "assistive technolog*" OR "ambient technolog*" OR "active" OR "assisted living" OR "active assisted living" OR "ambient assisted living" OR "gaming" OR "game" OR "games" OR "virtual companion*" OR "virtual realit*" OR "augmented realit*" OR "mixed realit*") OR AB("robot*" OR "telerobot*" OR "decision support*" OR "decision aid*" OR "smart home*" OR "assistive technolog*" OR "ambient technolog*" OR "active" OR "assisted living" OR "active assisted living" OR "ambient assisted living" OR "gaming" OR "game" OR "games" OR "virtual companion*" OR "virtual realit*" OR "augmented realit*" OR "mixed realit*") OR KW("robot*" OR "telerobot*" OR "decision support*" OR "decision aid*" OR "smart home*" OR "assistive technolog*" OR "ambient technolog*" OR "active" OR "assisted living" OR "active assisted living" OR "ambient assisted living" OR "gaming" OR "game" OR "games" OR "virtual companion*" OR "virtual realit*" OR "augmented realit*" OR "mixed realit*") | **174,364** |
| **S2** | TI("smart" OR "intelligent" OR "adaptive" OR "predict*" OR "autonomous*" OR "self learning*" OR "data driven*") OR AB("smart" OR "intelligent" OR "adaptive" OR "predict*" OR "autonomous*" OR "self learning*" OR "data driven*") OR KW("smart" OR "intelligent" OR "adaptive" OR "predict*" OR "autonomous*" OR "self learning*" OR "data driven*") | **390,991** |
| **S1** | MH "Nursing Homes" OR MH "Nursing Home Patients" OR MH "Gerontologic Care" OR TI("home of the aged" OR "homes for the aged" OR "old age home*" OR "healthy aging" OR "active aging" OR "healthy ageing" OR "active ageing" OR "silver econom*" OR (("elder*" OR "eldest" OR "geriatri*" OR "gerontol*" OR "old age*" OR "oldest old*" OR "senior*" OR "senium" OR "very old*" OR "septuagenarian*" OR "octagenarian*" OR "octogenarian*" OR "nonagenarian*" OR "centarian*" OR "centenarian*" OR "supercentenarian*" OR "older people" OR "older subject*" OR "older patient*" OR "older age*" OR "older adult*" OR "older man" OR "older men" OR "older male*" OR "older woman" OR "older women" OR "older female*" OR "older population*" OR "older person*" OR "ageing*" OR "aging*" OR "amenti*" OR "alzheimer*" OR "boomer*" OR "later life*") AND ("care" OR "assisted liv*" OR "rehabilitation" OR "residential*" OR "community-dwelling*" OR "home-dwelling*" OR "independent living*" OR "nursing home*"))) OR AB("home of the aged" OR "homes for the aged" OR "old age home*" OR "healthy aging" OR "active aging" OR "healthy ageing" OR "active ageing" OR "silver econom*" OR (("elder*" OR "eldest" OR "geriatri*" OR "gerontol*" OR "old age*" OR "oldest old*" OR "senior*" OR "senium" OR "very old*" OR "septuagenarian*" OR "octagenarian*" OR "octogenarian*" OR "nonagenarian*" OR "centarian*" OR "centenarian*" OR "supercentenarian*" OR "older people" OR "older subject*" OR "older patient*" OR "older age*" OR "older adult*" OR "older man" OR "older men" OR "older male*" OR "older woman" OR "older women" OR "older female*" OR "older population*" OR "older person*" OR "ageing*" OR "aging*" OR "amenti*" OR "alzheimer*" OR "boomer*" OR "later life*") AND ("care" OR "assisted liv*" OR "rehabilitation" OR "residential*" OR "community-dwelling*" OR "home-dwelling*" OR "independent living*" OR "nursing home*"))) OR KW("home of the aged" OR "homes for the aged" OR "old age home*" OR "healthy aging" OR "active aging" OR "healthy ageing" OR "active ageing" OR "silver econom*" OR (("elder*" OR "eldest" OR "geriatri*" OR "gerontol*" OR "old age*" OR "oldest old*" OR "senior*" OR "senium" OR "very old*" OR "septuagenarian*" OR "octagenarian*" OR "octogenarian*" OR "nonagenarian*" OR "centarian*" OR "centenarian*" OR "supercentenarian*" OR "older people" OR "older subject*" OR "older patient*" OR "older age*" OR "older adult*" OR "older man" OR "older men" OR "older male*" OR "older woman" OR "older women" OR "older female*" OR "older population*" OR "older person*" OR "ageing*" OR "aging*" OR "amenti*" OR "alzheimer*" OR "boomer*" OR "later life*") AND ("care" OR "assisted liv*" OR "rehabilitation" OR "residential*" OR "community-dwelling*" OR "home-dwelling*" OR "independent living*" OR "nursing home*"))) | **130,265** |

**Supplementary Section C: Additional comments on screening process**

We determined if papers were relevant for this scoping review based on three inclusion criteria: long-term care, Artificial Intelligence, Responsible Innovation. We first performed a title and abstract screening to select papers that meet all three main inclusion criteria. In a second round of full-text screening, we excluded records that discussed any of the three core search concepts only marginally and made an insufficient link *between* the three core search concepts.

In the following, some additional comments are provided to complement our explanation of the three core search concepts in the main paper.

Long-term care (LTC): eligible papers address technological systems or services that are (to be) used in the LTC for older adults, i.e. in the assistance given over an extended period of time to people who, as a result of aging and related conditions such as dementia, experience inabilities to perform tasks associated with everyday living (Kane et al., 1998; H. S. Kaye et al., 2017). These technologies can be used in institutionalized, community- or home-based care settings by older adults that receive some form of formal or informal care, and/or by their caregivers.

*Additional comment:* This excludes AI technologies focused on hospital(ized) care for older adults, AI technologies focused specifically on the diagnosis and/or treatment of illnesses such as stroke, cancer, diabetes, COPD, osteoporosis and multiple sclerosis, with dementia as an exception. Also, papers are excluded that address applications of AI that are not directly used by older adults and/or their caregivers/the care organisation, but rather by researchers in an academic setting, for instance.

Artificial Intelligence (AI): eligible papers provide information about the (semi-)autonomous decision-making capabilities of the addressed technologies, i.e. about the data-processing mechanisms that enable them to carry out certain tasks independently. Responsible AI innovation can only be properly assessed if clear explanations are provided about the role of AI in the article (Hagendorff, 2020).

*Additional comment:* In order to structure the screening of title-abstracts on this criterium, we distinguished between four categories of papers:

- papers that explicitly indicate that the respective technology is driven by AI by using terms such as “automated”, “autonomous”, “self-learning”, “machine learning” and “rule-based”, or by mentioning AI-based functionalities such as “natural language processing”, “speech recognition” or “computer vision” (label “AI-3”).
- papers from which the reviewer expects that AI is involved and some type of automated or autonomous decision-making or operation by technology is taking place, but which do not use terms like “Artificial Intelligence”, “automated”, “autonomous”, “self-learning”, “machine learning” and “rule-based” (label “AI-2”).
- papers that address technologies that are potentially driven by AI, without discussing the possible role of AI (label “AI-1”). For instance, these papers use adjective terms such as “intelligent”, “smart”, “adaptive”, “predictive”, “robot”, “ambient”, but according to the reviewer, it was not apparent why these adjectives apply.
- papers that clearly do not address AI technologies that are (to be) used by older adults and/or their caregivers (label “AI-0)

Papers were only included in the subsequent round of full-text screening if they were labelled as AI-3 or AI-2 by both reviewers and if they also met the other two inclusion criteria (long-term care and Responsible Innovation).

Responsible Innovation (RI): eligible papers report on recommendations for decisions in practice to foster the responsible design and/or implementation of AI technologies in LTC. For instance, eligible papers explicate how certain measures on the level of AI technologies’ design or implementation contribute to the ethical acceptability, sustainability and/or social desirability of these technologies (Von Schomberg, 2013) or to compliance with responsible AI principles such as transparency, justice and fairness (Jobin et al., 2019). Papers are excluded if they question *if* AI technologies can (or should) be responsibly used in LTC without discussing *how* they can be responsibly designed or implemented. Papers are also excluded when they discuss which RI issues should be addressed in context of a particular AI technology, without providing clues on how to address these issues at the level of the technology’s design or implementation. Further, papers are excluded if they solely assess the accuracy, usability or acceptability of technologies.

*Additional comment***:** It was found during title-abstract screening process that a fair number of identified papers apply the principle of privacy, which is recognized to be a critical issue in the research fields of responsible AI innovation (Jobin et al., 2019) and gerontechnology (Chung et al., 2016). Notwithstanding that these studies may all contribute to responsible AI innovation in LTC, the literature reviewers decided to exclude most of these papers from further analysis because they lack substantial depth on the importance of privacy in the context of AI and LTC or because they reflect the common assumption that the use of certain methods for monitoring older adults is less intrusive than others; e.g. sensor-based monitoring as compared to camera-based monitoring or technological monitoring as compared to traditional methods such as direct observation (Grigorovich & Kontos, 2020).
